# Platelet-derived extracellular vesicles promote endothelial dysfunction in sepsis by enhancing neutrophil extracellular traps

**DOI:** 10.1186/s12865-023-00560-5

**Published:** 2023-08-09

**Authors:** Meini Jiang, Weidong Wu, Yanmei Xia, Xiuzhe Wang, Jifang Liang

**Affiliations:** 1 Department of critical care medicine, Tongji Shanxi Hospital, Shanxi Bethune Hospital, Shanxi Academy of Medical Sciences, Third Hospital of Shanxi Medical University, Taiyuan, 030032 China; 2 Tongji Hospital, Tongji Medical College, Huazhong University of Science and Technology, Wuhan, 430030 China

**Keywords:** Sepsis, Endothelial dysfunction, Platelet-derived extracellular vesicles (PEVs), Neutrophil extracellular traps (NETs)

## Abstract

**Background:**

The role of platelet-derived extracellular vesicles (PEVs) in the development of sepsis was investigated in this study.

**Methods:**

After collection of blood samples from sepsis patients and normal volunteers, the extracellular vesicles (EVs) were separated, followed by the isolation of PEVs from the blood of rats. Next, a sepsis rat model was constructed by cecal ligation and puncture (CLP), and rats received tail vein injection of PEVs to explore the role of PEVs in sepsis. Subsequently, nanoparticle tracking analysis (NTA) and transmission electron microscopy (TEM) were adopted to determine the diameter of EVs and observe the morphology of PEVs, respectively; flow cytometry to detect the percentage of CD41-and CD61-positive EVs in isolated EVs; and ELISA to assess neutrophil extracellular trap (NET) formation, endothelial function injury-related markers in clinical samples or rat blood and serum inflammatory factor level.

**Results:**

Compared with normal volunteers, the percentage of CD41- and CD61-positive EVs and the number of EVs were significantly elevated in sepsis patients. Moreover, sepsis patients also presented notably increased histone H3, myeloperoxidase (MPO), angiopoietin-2 and endocan levels in the blood, and such increase was positively correlated with the number of EVs. Also, animal experiments demonstrated that PEVs significantly promoted NET formation, mainly manifested as up-regulation of histone H3, high mobility group protein B1 (HMGB1), and MPO; promoted endothelial dysfunction (up-regulation of angiopoietin-2, endocan, and syndecan-1); and stimulated inflammatory response (up-regulation of interleukin (IL) -1β, IL-6, tumor necrosis factor (TNF)-α, and monocyte chemoattractant protein (MCP) -1) in the blood of sepsis rats.

**Conclusion:**

PEVs aggravate endothelial function injury and inflammatory response in sepsis by promoting NET formation.

## Introduction

Sepsis, a common disease, is defined as a life-threatening organ dysfunction induced by dysregulated host response to infection [1]. Currently, sepsis has become a fatal threat to global public health. It is estimated that sepsis affects 30 million people and causes 6 million deaths each year worldwide [2]. Endothelial dysfunction is the main pathophysiological characteristic during the development of sepsis [3]. More concretely, exogenous pathogen-associated molecular patterns (PAMPs) and endogenous damage-associated molecular patterns (DAMPs) trigger endothelial activation during bacterial, fungal, or viral infections and may impair endothelial structure and function [4]. Study has shown that normal endothelial responses are the most effective way to limit bacterial transmission, coordinate leukocyte recruitment and promote bacterial clearance. However, excessive or persistent changes in endothelial phenotype may result in impaired microcirculatory blood flow, tissue hypoperfusion, and life-threatening organ failure [5]. Therefore, understanding the pathways of sepsis causing endothelial dysfunction is a promising way to limit sepsis-induced organ failure.

Neutrophils are the most abundant leukocytes in the body, serve as the first line of defense against infection, and play a crucial part in the innate immune response during sepsis through the secreting regulatory cytokines, chemokines, leukotrienes and endocytosing pathogens [6]. It’s worth noting that the imbalance of neutrophils regulation is directly related to the mortality of sepsis [6]. Meanwhile, neutrophils kill ingested pathogens through phagocytosis, generation of reactive oxygen species (ROS), release of protease-enriched granules and formation of neutrophil extracellular traps (NETs) [7]. NETs are characterized by complex structures composed of nuclear chromatin, histones, granular antimicrobial proteins and some cytoplasmic proteins. As a second bactericidal mechanism of neutrophils, NETs are able to physically trap bacteria and promote interactions between bacteria and antimicrobial effectors [8]. Notably, NETs may be a double-edged sword because excessive NET formation during sepsis can trigger multiple organ dysfunction [9]. Study manifested that though NET contributed to pathogen clearance, excessive NET formation would promote inflammation and tissue damage in sepsis [10]. Moreover, NETs have been reported to induce endothelial damage mediated by extracellular histones, neutrophil granule proteins, and tangled extracellular DNA in the lung [11]. Shortly speaking, NET over-formation is a potential factor for endothelial injury.

As one of the important components of the blood, platelets are not only the most crucial element in hemostasis and thrombosis, but also mediators of the body’s comprehensive inflammatory response [12]. Besides, accumulating in vitro and in vivo evidence suggests that platelets may contribute to the NET formation through exosomes and by forming platelet–platelet and platelet-neutrophil aggregates [13]. Nevertheless, neither the process nor the mechanism of platelet-activated NET formation is clearly expounded in sepsis. Recently, it has been pointed out that platelet-derived extracellular vesicles (PEVs) promote NET formation during septic shock [14]. A majority of extracellular vesicles (EVs) in peripheral blood are derived from platelets. Previous study has shown that lipid vesicles and PEVs, characterized with stable phospholipid bilayer structures and carrying platelet contents (nucleic acid, surface protein, and organelles), can play an important role in a variety of physiological and pathological conditions [15]. A review by Puhm et al. stated that over-activated platelets and increased PEV circulation in the blood were associated with high inflammatory responses, thrombosis, and multiple organ damage in COVID-19 patients [16]. Xu et al. claimed that circulating plasma EVs from sepsis mice induced inflammation through a mechanism dependent on microRNA and TLR7 [17]. Briefly, PEVs might induce inflammation during sepsis. Recently, Jiao et al. discovered that platelet-derived exosomes regulated Akt/mTOR-related autophagic pathway with carrying HMGB1 or miRNA, thereby inducing the formation of NET and the occurrence of lung injury [14]. Also, Tang et al. demonstrated that PEVs enhanced the proangiogenic potential of adipose-derived stem cell (ADSC)s in mouse hindlimb ischaemia mainly by promoting ADSC proliferation, migration, anti-apoptosis ability and paracrine secretion [18]. However, the role of PEVs in NET formation, inflammatory response, and endothelial dysfunction in sepsis still remains to be explored. In this study, we revealed a significant correlation between PEVs and NET formation in collected clinical blood samples of sepsis patients. Additionally, after constructing an animal model through cecal ligation and puncture (CLP), we further validated the correlation of PEVs with NET formation, endothelial injury, and inflammatory response in sepsis rats. Briefly speaking, the objective of this study was to provide new directions and data support for clinical treatment of sepsis-induced endothelial injury.

## Materials and methods

### Clinical sample collection

This study was approved by the Human and Animal Ethics Committee of Shanxi Bethune Hospital (YXLL-2019-84) and conducted in accordance with the approved guidelines. All patients were informed and voluntarily signed the informed consent form. Whole blood samples were collected from patients with sepsis (Sepsis group, n = 30) treated in Shanxi Bethune Hospital and normal volunteers (Normal group, n = 30) from June 2020 to December 2020 and placed in collecting tube with/without an anticoagulant—ethylenediaminetetraacetic acid (EDTA). The clinical baseline information of all patients was collected. Patients were included in this study if they were diagnosed with sepsis according to *The Third International Consensus Definitions for Sepsis and Septic Shock (Sepsis-3)* [Sequential Organ Failure Assessment (SOFA) ≥ 2, systolic blood pressure ≤ 100 mm Hg, respiratory rate ≥ 22 times/min] [19]. As for exclusion criteria, patients were excluded if they (1) suffered from heart, lung, kidney dysfunction or other immune system diseases; (2) had blood system diseases, coagulation disorders, mental disorders; (3) or were pregnant.

### Isolation of platelet-derived extracellular vesicles

The clinical blood sample collected from human was centrifuged at 120 g for 25 min to obtain platelet-rich plasma (PRP). The PRP was centrifuged again at 750 g for 15 min to obtain the platelet, then washed with buffer (0.12 M sodium chloride, 0.0129 M trisodium citrate, 0.03 M D-glucose, pH 6.5). Subsequently, the washed platelets were suspended in physiological saline and adjusted to a final concentration of 5 × 10^9^ platelets/ml. The platelet suspension was activated with thrombin (0.5 NIH Units/ml, 9002-04-4, Sigma-Aldrich, MO, USA) for 15 min at 37 °C as previously described [20]. Three Sprague Dawley (SD) rats (Weitonglihua, Beijing, China) were fasted for 12 h. Next, venous blood was drawn from the tail vein, collected into a test tube containing 3.8% trisodium citrate anticoagulant, centrifuged at 600 g for 15 min to obtain PRP. Then platelets were collected from PRP by centrifugation at 2000 g for 15 min, resuspended using Tyrode solution, and activated with 5 µg/mL collagen I at 37 °C for 1 h. After activating for 90 min, the solution was centrifuged at 2000 g for 15 min to remove the platelets. After that, the platelets were removed by a centrifugation step (3220 × g, 10 min, 20 °C) two times. EVs were then isolated using ExoQuick™ precipitation solution (EXOQ5A-1, System Biosciences, CA, USA) according to the instructions.

Subsequently, the morphology of PEVs was observed by a transmission electron microscopy (TEM, HT7700, HITACHI, Japan). The EV (10 µL) solution was placed on a formvar coated 400 mesh copper grid for 5 min of incubation, and the excess liquid was discarded. Then, uranyl acetate was added to the mesh for negative staining for 1 min, with excess liquid removed, and ultimately, the treated sample was observed at 100 kV through TEM. The size of the EV was tested via a NanoSight LM10-HS (NanoSight Ltd, Amesbury, United Kingdom) equipped with a 638 nm laser. Firstly, the sample was diluted to 1 mL with PBS, then the diluted sample was transferred into the sample chamber with a sterile syringe until the liquid reached the chamber outlet. Nanoparticles were irradiated by laser, and their motion under Brownian Motion is captured for 60 s. This process was repeated 5 times and 5 videos were acquired from each per sample. After that, data analysis was performed based on high-resolution particle size distribution profiles and concentration measurements of EVs provided by nanoparticle tracking analysis (NTA) software.

### Flow cytometry

The percentages of PEVs (CD41 + or CD61 + PEVs) in isolated EVs were measured by flow cytometry referring to previous studies [21]. Briefly, 100 µL EVs were incubated with 5 µL FITC-conjugated CD41 (11-0411-82, eBioscience, San Diego, CA, USA) or isotype control (11-4301-82, eBioscience, San Diego, CA, USA), APC-conjugated CD61 (17-0619-42, eBioscience, San Diego, CA, USA) or the respective isotype control (17-4714-82, eBioscience, San Diego, CA, USA) for 30 min in the dark at room temperature. After that, the sample was added with 500 µl PBS and centrifugated at 20,000 g at 4 °C for 30 min. Then sample was resuspended in 500 µl PBS for analysis using FACSymphony A5 (Becton and Dickinson, NJ, USA) following gating strategy. Specifically, Flow Cytometry Sub-micron Particle Size Reference Kit (diameters ranging from 0.02 to 2.0 μm, F13839, Invitrogen, USA) were used to determine the relative size range of EVs according to the kit instructions by comparing the forward scattered signals of EVs and reference microspheres. A threshold gate was set on the forward scatter (FSC)/side scatter (SSC) dot plot to effectively remove unwanted events and reduce noise and the PEVs distribution region of 0.1–0.2 μm was delineated as the gate. The percentages of CD41 + or CD61 + PEVs were analyzed using FlowJo software (Tree Star, Inc., Ashland, OR, USA). The detecting volume was 500 µl, and the flow rate was set to 25 µl/min. Finally, the number of PEVs per µL was calculated using the formula of PEVs/µL = (number of CD41 positive events in flow cytometry×dilution ratio)/sample volume.

### Establishment and treatment of sepsis rat model

A sepsis rat model was established using cecal ligation and puncture (CLP) described by previous studies [22, 23]. Before surgery, all SD rats were fasted for 24 h but had free access to drinking water. Then, they were anesthetized with intraperitoneal injection of 1% pentobarbital (30 mg/kg) [24], and the degree of anesthesia was determined through the Pinch-Toe Test. Upon general anesthesia, the cecum of rats were exposed through laparotomy. The cecum was ligated at the position of its 1/2 and punctured once using a 21-gauge needle in the area with the fewest blood vessels. After extrusion of a small amount of the content from the perforation site, the cecum was put back and the wound was sutured. After surgery, 30 mL/kg of saline was injected subcutaneously for fluid replacement. Subsequently, 24 male SD rats (weighing 180 ~ 220 g) aged 6 to 8 weeks were randomly divided into three groups (n = 8): Sham group, CLP group, and CLP + PEVs group. In the Sham group, the cecum of rats were exposed and flipped, but not ligation and puncture. In the CLP group, rats were subjected to CLP to induce a sepsis model. As for the CLP + PEVs group, rats were administered PEVs (1 × 10^8^) via the tail vein. 30 min later, a sepsis rat model was constructed through CLP. After different treatments for 24 h, the rats in each group were anesthetized with intraperitoneal injection of 1% pentobarbital (30 mg/kg). The blood samples were collected into the collection tubes with/without EDTA anticoagulant. The rats were euthanized at the end of the study by deep intraperitoneal anesthesia with 100 mg/kg pentobarbital [25].

### ELISA

The clinical blood samples and rat blood samples collected with or without anticoagulant were centrifuged at 4 ℃ at 2000 g. After 10 min, the supernatant was collected to obtain serum or plasma. According to the instructions of manufacturers, corresponding ELISA kits (Wuhan Cusabio Co., Ltd., China), were applied to determine the levels of histone H3 (#72,856, Cell Signaling Technology, USA), high mobility group protein B1 (HMGB1, CSB-E08223h, CSB-E08224r), and myeloperoxidase (MPO, CSB-E08721h, CSB-E08722r) in the serum as well as the levels of angiopoietin-2 (CSB-E04500h, CSB-E07304r), endocan (ml038029, ml059536, Shanghai Enzyme-link Biotech Co., Ltd., China), syndecan-1 (CSB-E17115r), interleukin (IL) -1β (CSB-E08055r), IL-6 (CSB-E04640r), tumor necrosis factor (TNF) -α (CSB-E11987r), and monocyte chemoattractant protein (MCP) -1 (CSB-E07429r) level in plasma.

### Statistical analysis

Measurement data were expressed as mean ± standard deviation (SD), and independent sample t test was employed for comparison between two groups, one-way analysis of variance for comparison among multiple groups. Counting data were represented by n (%), and chi-square test was used for comparison between groups. Graphpad prism 9.0 software was adopted for plotting. SPSS 26.0 and Graphpad prism 9.0 were utilized for statistical analysis, and multivariate logistic regression models for assessing the association of EVs/µL with the histone H3, MPO, angiopoietin-2, and endocan levels. *P* < 0.05 indicated significant differences.

## Results

### Characterization of platelet-derived extracellular vesicles in sepsis patients and normal volunteers

**Fig. 1:**
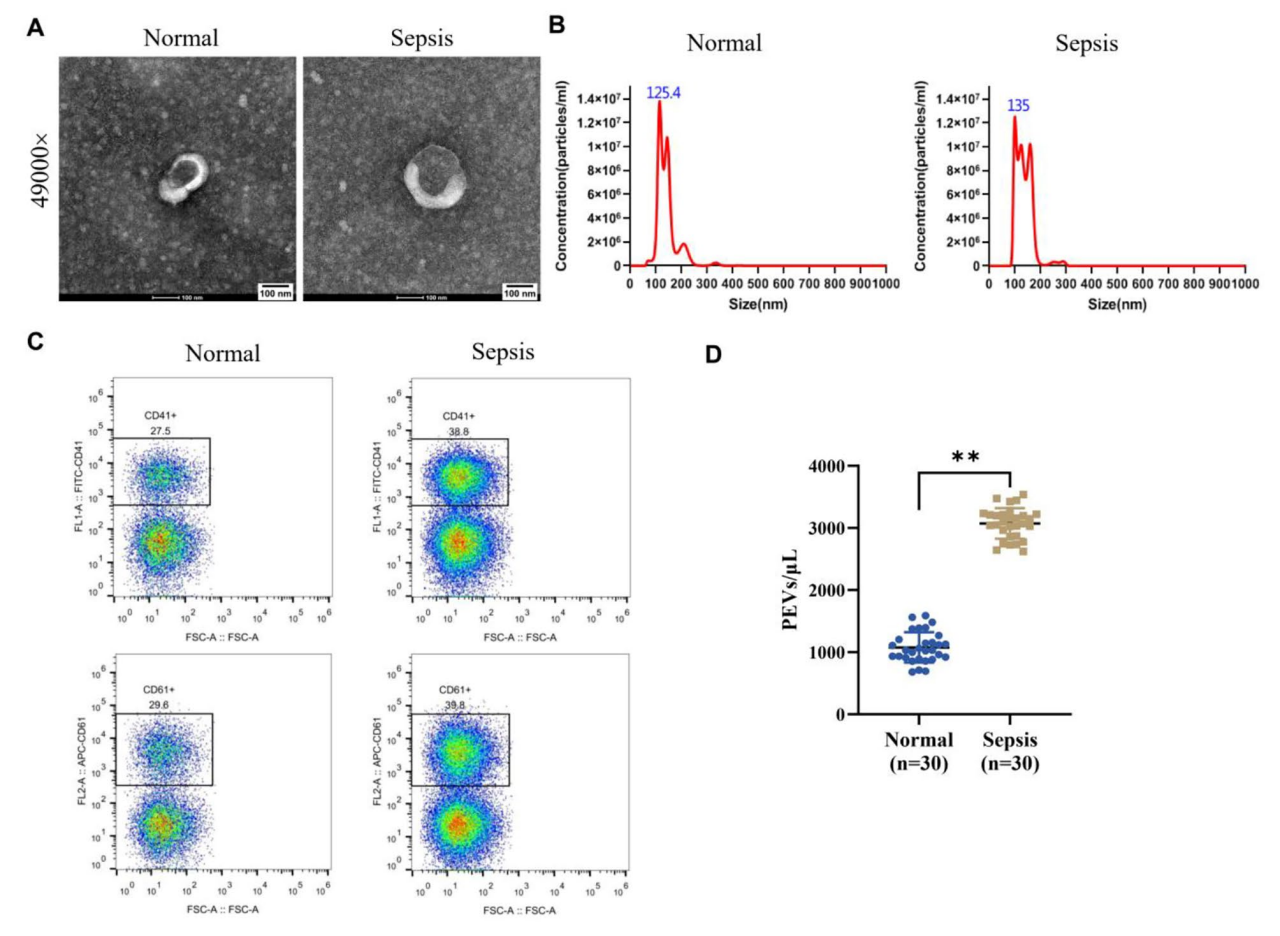
Characterization of platelet-derived extracellular vesicles (PEVs) in sepsis patients and healthy volunteers. **A**: The morphology of extracellular vesicles (EVs) isolated from clinical blood samples was observed by transmission electron microscopy (TEM); **B**: The diameter of EVs isolated from clinical blood samples was analyzed by nanoparticle tracking analysis (NTA); **C**: The proportion of EVs with surface markers CD41 + or CD61 + in isolated EVs was detected by flow cytometry. **D**: Comparison of the number of PEVs (CD41 + EVs) in clinical blood samples from normal healthy volunteers and patients with sepsis, **P < 0.01 vs. Normal group. Normal (n = 30) and Sepsis (n = 30) group, EV: extracellular vesicle; TEM, transmission electron microscopy; NTA, nanoparticle tracking analysis

Whole blood samples and clinical features were collected from 30 sepsis patients and 30 normal healthy volunteers to explore the role of PEVs in sepsis (Table 1). The EVs were isolated from the platelet free plasma prepared from blood collected from clinical samples in anticoagulant, followed by observation of classical vesicular morphology under TEM (Fig. 1A). Subsequently, NTA analysis revealed the mean diameters of EVs of 125.4 nm (1.4 × 10^7^ particles/mL) and 135 nm (1.2 × 10^7^ particles/mL), respectively, suggesting successful isolation and acquisition of EVs (Fig. 1B). Further detection of platelet-specific surface markers (CD41 and CD61) in EVs by flow cytometry indicated that, compared with the Normal group, the percentage of CD41 + or CD61 + EVs and the number of CD41 + EVs were remarkably higher in the Sepsis group (*P* < 0.01, Fig. 1C/D).

**Table 1:** Demographic and clinical features of sepsis patients and normal volunteers

Characteristic	Normal group (n = 30)	Sepsis group (n = 30)	*P* value
**Sex**			1.000
Female, n (%)	3(10.00)	3(10.00)	
Male, n (%)	27(90.00)	27(90.00)	
**Age**, years, Mean ± SD	49.13 ± 7.22	51.60 ± 6.69	0.175
**BMI**, kg/m^2^, Mean ± SD	25.38 ± 3.69	22.10 ± 2.75	< 0.001
**Cause of sepsis**			-
Infection, n (%)	-	24(80.00)	
Non-infection, n (%)	-	6(20.00)	
**Hematologic**, Mean ± SD			
WBC, 10^9^/L	12.41 ± 3.93	6.39 ± 3.93	< 0.001
RBC, 10^9^/L	3.62 ± 1.20	4.25 ± 0.84	0.022
Neutrophils, 10^9^/L	3.54 ± 1.26	11.41 ± 3.08	< 0.001
Platelets, 10^9^/L	177.06 ± 26.92	143.36 ± 17.07	< 0.001
CRP, mg/dL	7.05 ± 2.15	19.67 ± 5.38	< 0.001
AST, U/L	142.86 ± 19.58	31.61 ± 6.13	< 0.001
ALT, U/L	132.17 ± 14.00	35.12 ± 9.59	< 0.001
Albumin, g/L	28.51 ± 4.26	46.55 ± 7.35	< 0.001
Bilirubin, µmol/L	17.22 ± 3.46	14.93 ± 2.72	0.006
Blood glucose, mmol/L	19.26 ± 4.48	5.09 ± 1.98	< 0.001
Creatinine, µmol/L	110.24 ± 22.56	91.89 ± 18.26	0.001
Lactic acid, mmol/L	6.39 ± 3.22	1.42 ± 0.56	< 0.001
SOFA score, Mean ± SD	-	7.17 ± 2.63	-
APACHE II, Mean ± SD	-	19.40 ± 5.20	-
**Infection site**, n (%)			-
Respiratory system	-	13(43.33)	
Gastrointestinal tract	-	9(30.00)	
Bloodstream infection	-	5(16.67)	
other	-	3(10.00)	

WBC, white blood cell; CRP, C-reactive protein; APACHE II, Acute physiology and chronic health evaluation II; SOFA, Sequential Organ Failure Assessment; SD, standard deviation

### Neutrophil extracellular trap formation and endothelial injury in sepsis patients

**Fig. 2:**
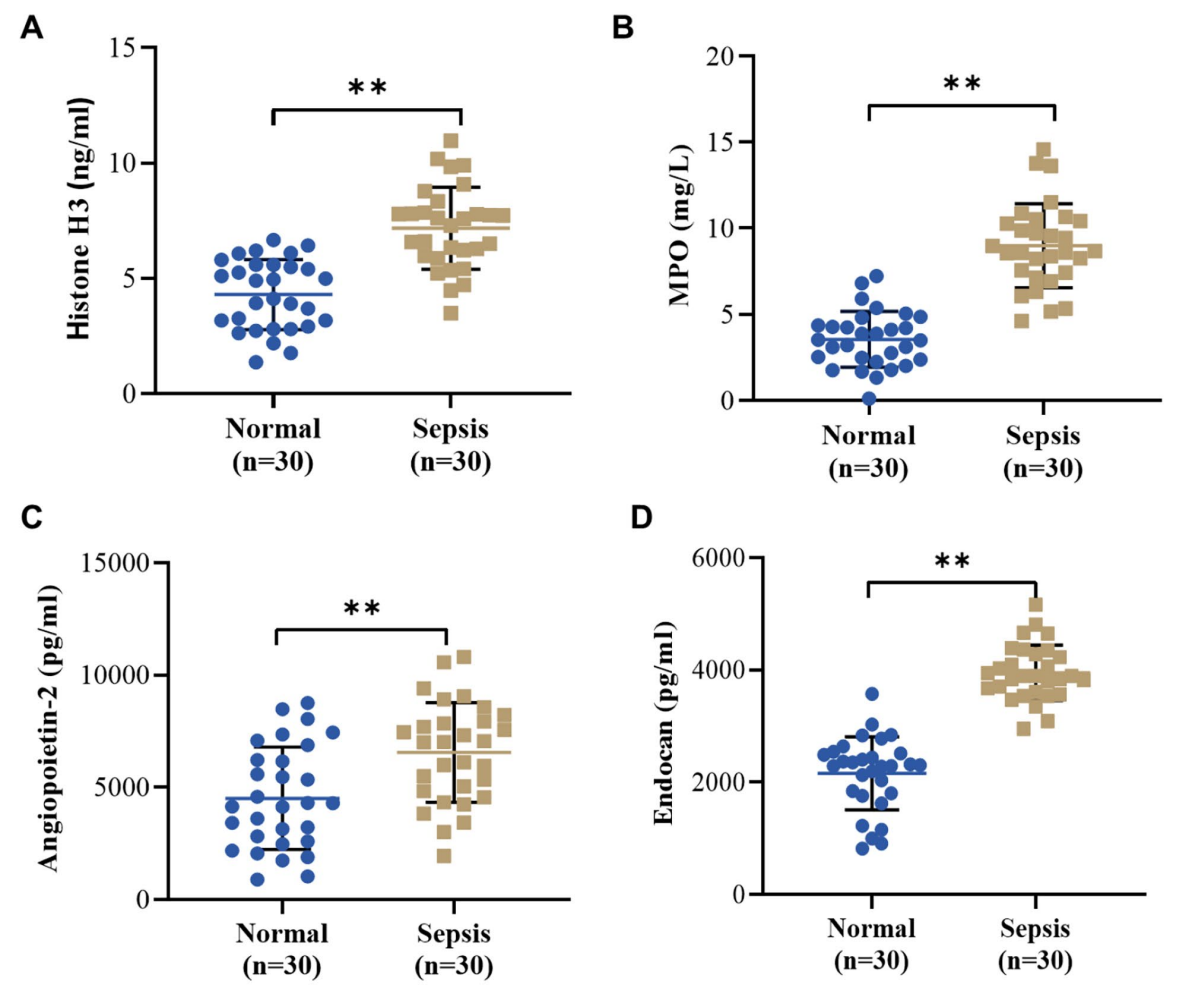
Biomarkers of Neutrophil extracellular traps (NETs) formation and endothelial injury in sepsis patients. **A**-**D**: ELISA was used to detect the level of histone H3 (**A**), MPO (**B**)--markers of NET formation, as well as angiopoietin-2 (**C**) and endocan (**D**)--markers of endothelial injury in clinical plasma and serum samples from the Normal (n = 30) and Sepsis (n = 30) group, **P < 0.01 and *P < 0.05 vs. Normal group. MPO, myeloperoxidase; NET, Neutrophil extracellular trap

To identify NET formation and endothelial injury in sepsis patients, the levels of NET formation markers (histone H3 and MPO) and endothelial dysfunction and injury markers (angiopoietin-2 and endocan) in clinical blood samples were examined. In brief, compared with normal volunteers, sepsis patients presented significantly elevated levels of histone H3 (4.303 ± 1.516 ng/mL vs. 7.181 ± 1.781 ng/mL, *P* < 0.01) and MPO (3.545 ± 1.625 ng/L vs. 8.983 ± 2.445 ng/L, *P* < 0.01) in the serum (Fig. 2A/B) and evidently increased levels of angiopoietin-2 (4512 ± 2284 pg/ml vs. 6552 ± 2220 pg/mL, *P* < 0.01) and endocan (2158 ± 650.3 pg/mL vs. 3948 ± 494.7 pg/mL, *P* < 0.01) in the plasma (Fig. 2C/D). The above outcomes suggested that NET formation and endothelial damage were significant in sepsis patients.

### Correlation of the number of platelet-derived extracellular vesicles with neutrophil extracellular trap formation and endothelial damage

**Fig. 3:**
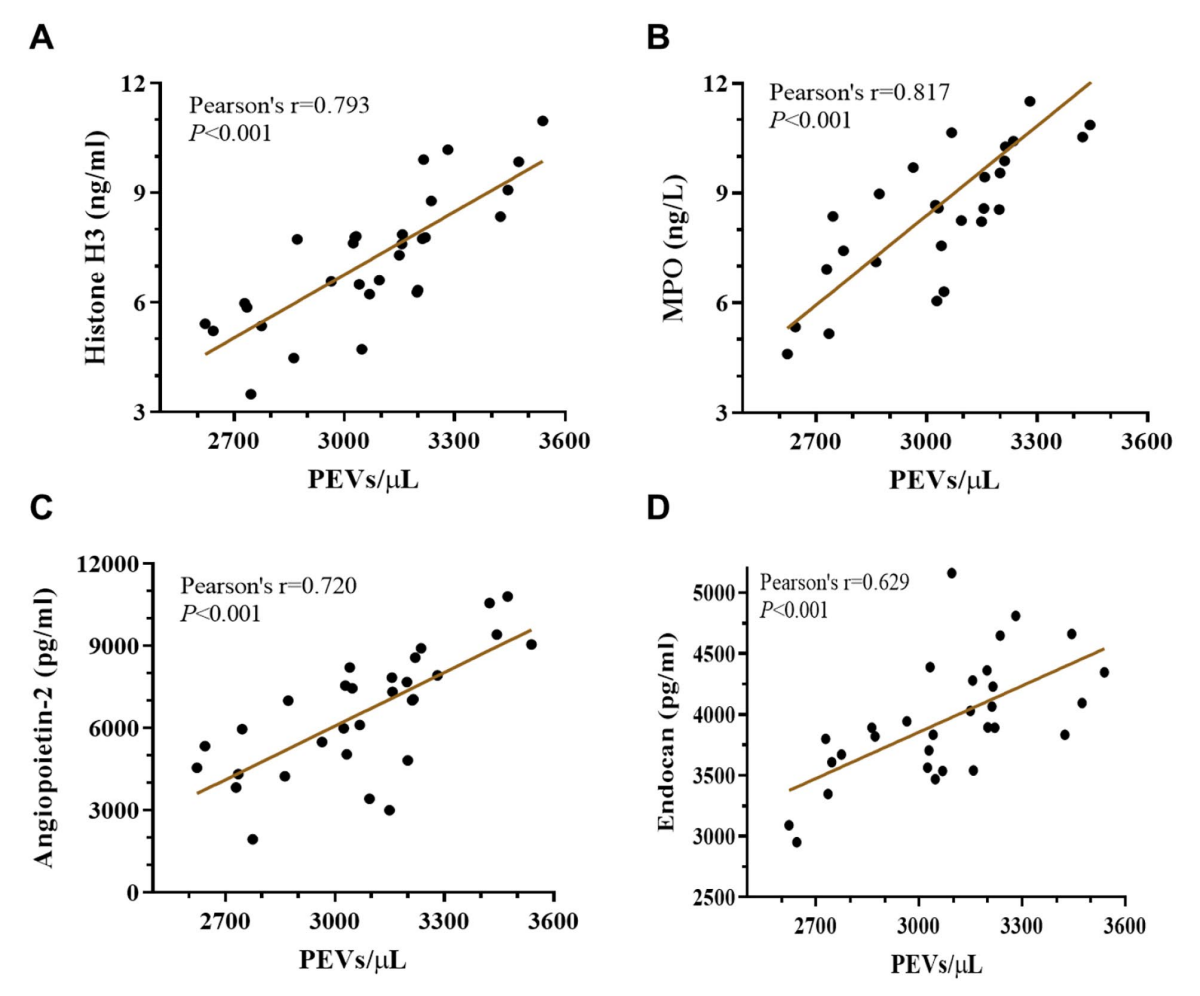
Correlation of the number of PEVs with markers of NET formation and endothelial injury. **A**-**D**: Linear regression assessed the correlation between the number of PEVs and histone H3 (**A**), MPO (**B**), angiopoietin-2 (**C**) and endocan (**D**) level in sepsis patients (n = 30). PEV, platelet-derived extracellular vesicles; MPO, myeloperoxidase

Previous results in our study demonstrated a noticeable increase in the number of PEVs, an obvious elevation in NET formation and a significant aggravation in endothelial injury in sepsis patients. To clarify whether NET formation and endothelial injury affected the number of PEVs, we evaluated the correlation of the histone H3, MPO, angiopoietin-2, endocan levels with the number of PEVs. To be specific, the number of PEVs in patients with sepsis were significantly positively correlated with the levels of serum histone H3 (Pearson’s r = 0.793, *P* < 0.001), MPO (Pearson’s r = 0.817, *P* < 0.001), angiopoietin (Pearson’s r = 0.720, *P* < 0.001) and endocan (Pearson’s r = 0.629, *P* < 0.001) (Fig. 3A-D). Therefore, the number of PEVs may be associated with NET formation and endothelial injury in sepsis.

### Characterization of platelet-derived extracellular vesicles in rat plasma

**Fig. 4:**
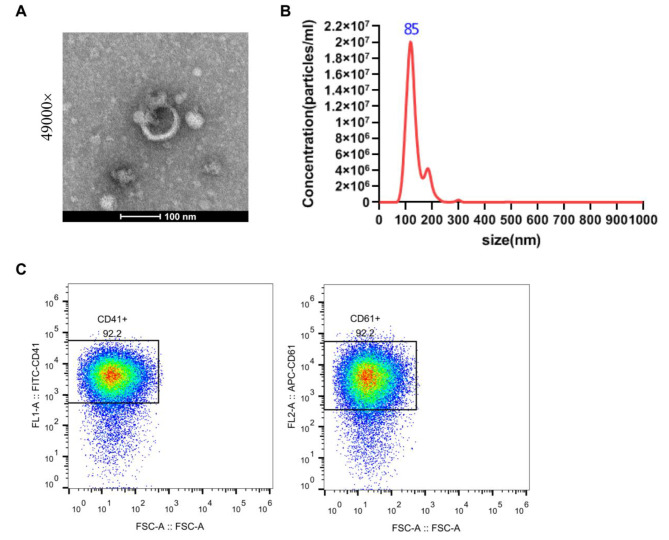
Characterization of PEVs in rat plasma. **A**: TEM was utilized to observe the morphology of PEVs in rat blood; **B**: NTA was employed to analyze the diameter of PEVs in rat blood; **C**: Flow cytometry was applied to detect the proportion of EVs and CD41- and CD61-positive PEVs. PEV, platelet-derived extracellular vesicles; TEM, transmission electron microscopy; NTA, nanoparticle tracking analysis

To further validate the role of PEVs in sepsis, EVs were isolated from rat blood and observed. Under TEM, a typical EV morphology could be observed (Fig. 4A). Moreover, NTA analysis showed an average diameter of 85 nm of EVs in rat blood (Fig. 4B). In addition, the isolated EVs reached 92.2% CD41- or CD61- positive rate revealed by flow cytometry (Fig. 4C). The above outcomes indicated successful isolation of PEVs from rat plasma.

### Platelet-derived extracellular vesicles promote neutrophil extracellular trap formation and induce endothelial function impairment in sepsis rats

**Fig. 5:**
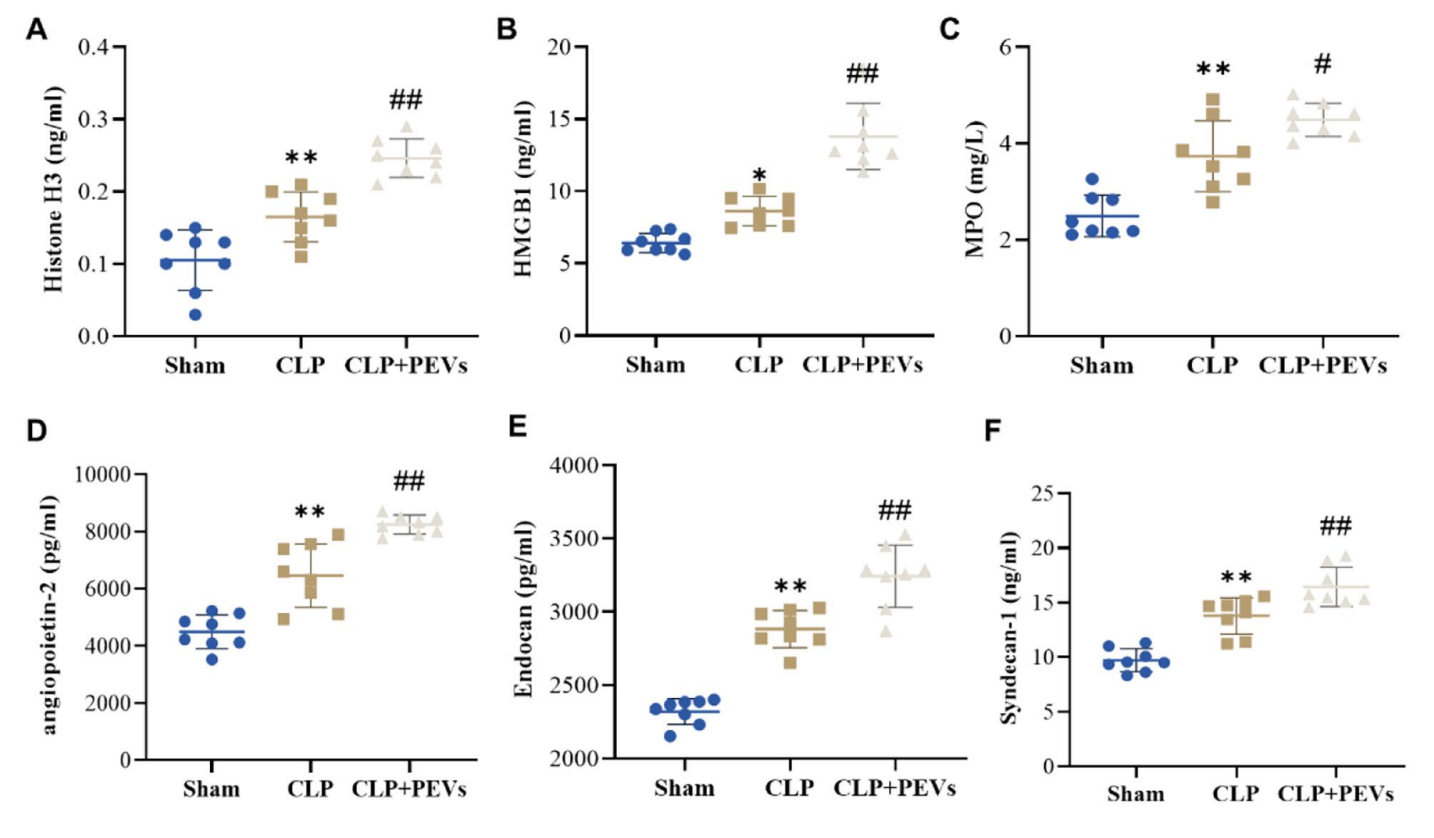
PEVs promote NET formation and endothelial function impairment in sepsis rats. A-F: The level of markers of NET formation: histone H3 (**A**), HMGB1 (**B**) and MPO (**C**), as well as level of markers of endothelial function impairment: angiopoietin-2 (**D**), endocan (**E**) and syndecan-1 (**F**) in the rats’ blood in each group (n = 8), were measured by ELISA, *P < 0.05 and **P < 0.01 vs. Sham group, #*P* < 0.05 and ##*P* < 0.01 vs. CLP group. NET, neutrophil extracellular traps; HMGB1, high mobility group protein B1; MPO, myeloperoxidase; CLP, cecal ligation and puncture

To further confirm the association of PEVs with NET formation and endothelial injury, a rat model of sepsis was constructed (CLP group) after treatment with PEVs. ELISA results showed that the levels of Histone H3, HMGB1, MPO, angiopoietin-2, endocan and syndecan-1 in the serum of rats in the CLP group were significantly higher than those in the Sham group (*P* < 0.05). Serum levels of the above markers were further increased in the PEV-treated CLP rats (*P* < 0.05) (Fig. 5A-F). Collectively, PEVs could promote NET formation and endothelial function impairment in sepsis rats.

### Platelet-derived extracellular vesicles aggravate the inflammatory response in sepsis rats

**Fig. 6:**
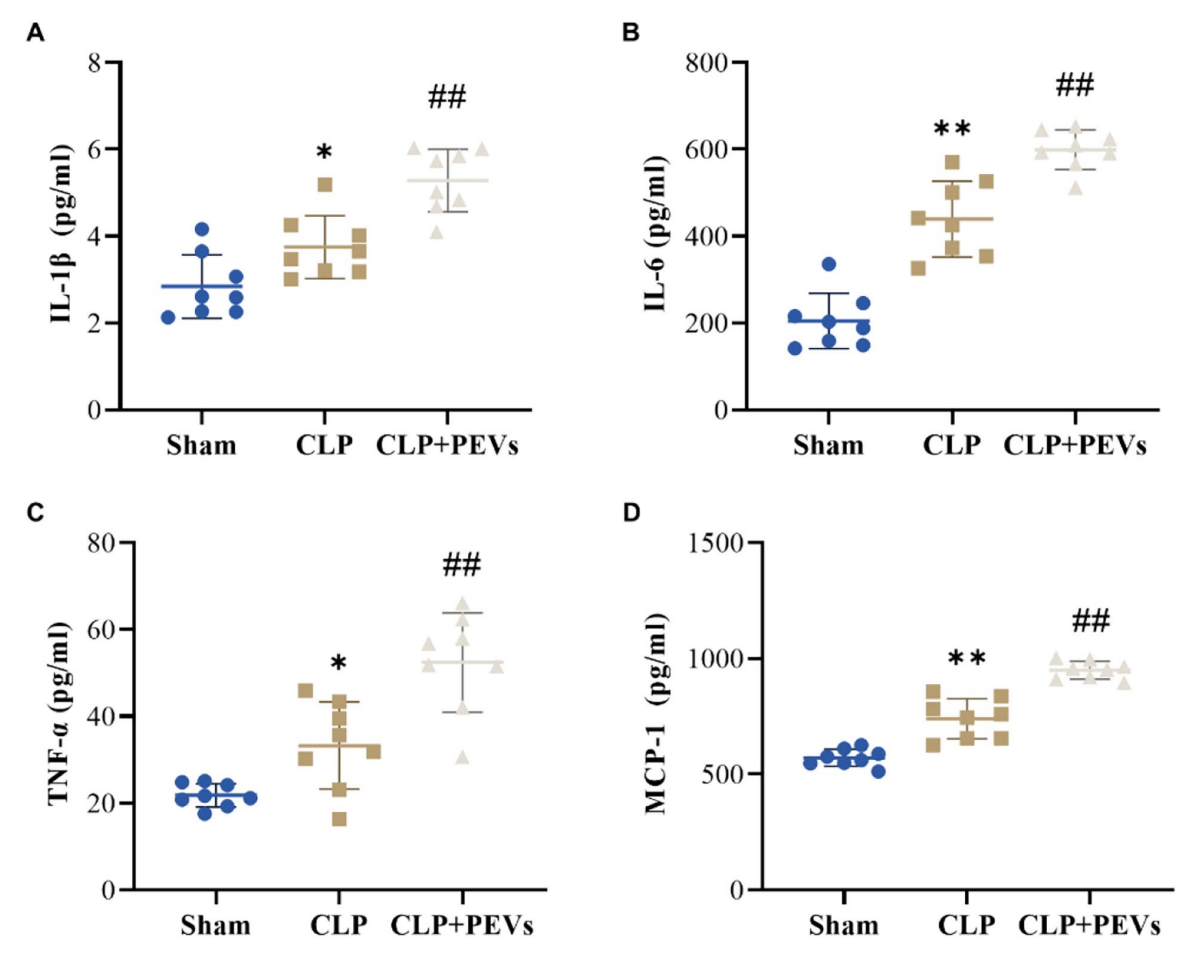
PEVs aggravate systemic inflammation in sepsis rats. A-D: The level of proinflammatory cytokines: IL-1β (**A**), IL-6 (**B**), TNF-α (**C**) and MCP-1 (**D**) in the rats’ blood of each group (n = 8) were measured by ELISA, *P < 0.05 and **P < 0.01 vs. Sham group, #*P* < 0.05 and ##*P* < 0.01 vs. CLP group. CLP, cecal ligation and puncture

As is known to all, inflammatory response plays a pivotal role in the development of sepsis. To clarify the role of PEVs in the inflammatory response of sepsis rats, the levels of inflammation-related factors in the blood of rats in each group were measured. The results of ELISA revealed enhanced expressions of IL-1β, IL-6, TNF-α and MCP-1 in the serum of rats in the CLP group relative to the Sham group (*P* < 0.05). Notably, the expressions levels of IL-1β (5.285 ± 0.7210 pg/mL vs. 3.749 ± 0.7230 pg/mL, *P* < 0.01), IL-6 (599.3 ± 45.40 pg/mL vs. 439.8 ± 87.29 pg/mL, *P* < 0.01), TNF-α (52.40 ± 11.43 pg/mL vs. 33.25 ± 10.03 pg/mL, *P* < 0.01) and MCP-1 (949.4 ± 38.92 pg/mL vs. 571.1 ± 36.71 pg/mL, *P* < 0.01) went up remarkably in the presence of PEVs (Fig. 6A–D). Overall, PEVs aggravated the inflammatory response by facilitating the secretion of pro-inflammatory cytokines in sepsis rats.

## Discussion

During sepsis (resulting from severe infection mostly with Gram-negative bacteria), platelets become overactivated and release PEVs containing numerous molecules and DAMPs, such as HMGB1, proinflammatory cytokines (IL-1β, TNF-α, IL-6) and chemokines (MCP-1). Activated platelets interact with circulating neutrophils forming platelet–neutrophil aggregates and HMGB1 present on PEVs causing activation of neutrophils, increased ROS production, and NET formation resulting in endothelial injury, thrombosis, and finally disseminated intravascular coagulation. Here, in this paper using blood samples from sepsis patients and controls, we have demonstrated elevated levels of platelet-derived EVs, platelet activation marker (HMGB1), surrogate markers of NET formation (histone H3 and MPO), and endothelial dysfunction and injury (angiopoietin-2 and endocan) in sepsis patients compared to controls. Further, using a rat model of sepsis and PEVs generated from thrombin-stimulated rat platelets, we demonstrated that PEVs promote NET formation (Histone H3, MPO, HMGB1), endothelial dysfunction (angiopoietin-2 (D), endocan (E) and syndecan-1) and aggravate the inflammatory response (IL-1, IL-6, TNF-α, and MCP-1) in sepsis.

EVs, as a key intercellular communicator, mediate molecular exchange between adjacent or distant cells and regulate cellular activities. Among the EVs of different cellular origin, PEVs are the most abundant ones in the blood. Previous study has shown that PEVs, carrying platelet contents (nucleic acids, surface proteins and organelles), play an important role in a variety of physiological and pathological conditions [15]. Moreover, PEVs are also described as a major contributor to infection [26]. In this paper, sepsis patients exhibited a much higher percentage of CD41- or CD61-positive EVs and a number of PEVs than normal volunteers. Previous studies reported a higher level of PEVs in HIV-infected patients; moreover, viral, parasitic or bacterial infections stimulate platelets to secrete more PEVs [27]. From the above findings, the rise in PEVs seems to be related to the host immune response.

The hyperinflammatory state together with impaired immune functions collectively leads to septic shock and lethality [28]. PEVs have been reported to play a pro-inflammatory role by secreting pro-inflammatory cytokines (IL-6) or carrying HMGB1 [29, 30]. Additionally, Danesh et al., revealed that PEVs mediate inflammatory responses by interaction with peripheral blood mononuclear cells (PBMCs), causing the secretion of pro-inflammatory chemokines and cytokines, and boosting T-cell responses [31]. Collectively, the regulation of inflammation by PEVs is diverse [32]. This study demonstrated that PEVs notably increased the expressions of proinflammatory factors in the blood of sepsis rats. Kong et al. consistently pointed out that PEVs significantly up-regulated pro-inflammatory factor level in HUVEC cells induced by PM 2.5, thereby inducing vascular endothelial injury [33]. The inflammatory response serves as an important signal while PEVs act as signaling mediators for sepsis development.

Neutrophils are the most important cells in the host’s natural defense against microorganisms. They play an important role in the innate immune response of sepsis through the secreting regulatory cytokines, chemokines, leukotrienes and endocytosing pathogens. It’s worth noting that the imbalance of neutrophils regulation is directly related to the mortality of sepsis [6]. NETs are characterized by complex structures composed of nuclear chromatin, histones, granular antimicrobial proteins and some cytoplasmic proteins. As a second bactericidal mechanism of neutrophils, NETs are able to physically trap bacteria and promote interactions between bacteria and antimicrobial effectors [8]. Studies manifested that though NET contributed to pathogen clearance, excessive NET formation would promote inflammation and tissue damage in sepsis [10]. Li et al. observed the elevation of the constituent components of NETs (histone H3) in patients with sepsis and septic shock [34]. Similarly, we also discovered that, in addition to great up-regulation in the plasma of sepsis patients and sepsis rats (CLP group), the NET formation markers (histone H3 and MPO) levels were positively correlated with the number of PEVs in sepsis patients. Moreover, a further up-regulation of histone H3 and MPO appeared in sepsis rats after injection of PEVs. Previous studies have revealed that the interactions of virus with platelet CLEC2 were shown to trigger the release of PEVs, activating neutrophils and inducing NETosis through heterocomplexes of TLR2 and CLEC5A [35, 36]. PEVs in sepsis might promote the formation of NETs by a similar mechanism. However, the mechanism of PEVs promoting the formation of NETs had not been explored in this study.

HMGB1 is a known inducer of autophagy. Studies have revealed that HMGB1 released by activated platelets as a mediator of NET formation could activates polymorphonuclear neutrophils, induces NET generation, neutrophil autophagy, and inhibit apoptosis [13, 37, 38]. Zhou et al. also claimed that platelet HMGB1 promotes platelet activation, thereby regulating platelet-neutrophil interactions and ROS production in neutrophils during sepsis [39]. Additionally, Jiao et al. revealed that PEVs induced activation of the intra-neutrophil Akt/mTOR-related autophagy pathway and subsequent NET formation via exosomal HMGB1 and/or miR-15b-5p and miR-378a-3p [14]. In our study, PEVs injection significantly increased the serum level of HMGB1 and markers of NETosis in sepsis rats, indicating that PEVs may promote the formation of NETs by regulating HMGB1. But the above conclusion was not verified by HMGB1 competitive antagonist BoxA [40], so it is not clear whether there are other regulatory mechanisms. Therefore, further experiments are needed to explore the mechanism of PEVs on NET formation.

Endothelial dysfunction and injury are the main pathophysiological characteristics of sepsis [3]. More interestingly, we found that the serum levels of angiopoietin-2 and endocan were positively correlated with the number of PEVs. Moreover, PEVs treatment further up-regulated the levels of angiopoietin-2 and endocan in septic rats’ plasma. Here, we have demonstrated the adverse effects of PEVs. Studies have shown that pro-inflammatory factors, PEVs and NETs cause endothelial damage and adverse results under a variety of infections [41, 42]. In a study by Widyaningrum et al., corneal endothelial cells treated with PEVs presented increased viability, an enhanced wound-healing rate, stronger proliferation markers, and an improved adhesion rate [43]. In our study, elevated endothelial function damage markers (angiopoietin-2 and endocan) levels were observed in the plasma of sepsis patients and rats, suggesting the activation and dysfunction of endothelium. This may be due to the up-regulation of inflammatory response, NET formation and endothelial dysfunction in clinical samples and animal models of sepsis.

To summarize, we demonstrated the correlation of up-regulation of PEVs with increase in surrogate markers of NET formation, severe inflammatory response and endothelial function impairment in sepsis patients using clinical blood samples and sepsis rat models. Nevertheless, we failed to reveal the specific mechanism by which PEVs regulate the inflammatory response in sepsis. Therefore, further exploration is required to more clearly reveal the influencing factors of sepsis progression and provide new ideas for the clinical treatment of sepsis.

## Conclusion

Collectively, PEVs are remarkably up-regulated in the plasma of sepsis patients and have a close association with the biomarkers of NETs and endothelial function impairment. Also, PEVs can greatly enhance biomarkers of NET formation, endothelial function impairment, and inflammatory response in sepsis rats. In brief, the presented evidence indicates that PEVs play an important role in promoting the progression of sepsis.

## Data Availability

The data used to support the findings of this study are available from the corresponding author upon request.

## Abbreviations

PEVs: Platelet-derived extracellular vesicles
CLP: Cecal ligation and puncture
NTA: Nanoparticle size tracking analysis
TEM: Transmission electron microscopy
NETs: Neutrophil extracellular traps
MPO: Myeloperoxidase
HMGB1: High mobility group protein B1
IL: Interleukin
TNF: Tumor necrosis factor
MCP: Monocyte chemoattractant protein
PAMPs: Pathogen-associated molecular patterns
DAMPs: Endogenous damage-associated molecular patterns
ROS: Reactive oxygen species
